# Praktische Aspekte bei der Anwendung von Topika in der geriatrischen Dermatologie

**DOI:** 10.1007/s00105-023-05187-6

**Published:** 2023-07-26

**Authors:** K. Therstappen, A. Eichner, J. Wohlrab

**Affiliations:** 1 Universitätsklinik und Poliklinik für Dermatologie und Venerologie, Martin-Luther-Universität Halle-Wittenberg, Ernst-Grube-Str. 40, 06120 Halle (Saale), Deutschland; 2 An-Institut für angewandte Dermatopharmazie (IADP), Martin-Luther-Universität Halle-Wittenberg, Weinbergweg 23, 06120 Halle (Saale), Deutschland

**Keywords:** Altershaut, Hautalterung, Altersdermatosen, Epidermis, Basispflege, Aged skin, Skin aging, Geriatric dermatoses, Epidermis, Basic skin care

## Abstract

Altersbedingte Veränderungen des Hautorgans beziehen in Abhängigkeit der intrinsischen Gegebenheiten und extrinsischer Einflussfaktoren alle kutanen Schichten ein. Das Ausmaß der seneszenten Veränderungen kann bei Hochaltrigen stark variieren, sodass eine individuelle Bewertung sinnvoll und häufig auch notwendig ist. Von besonderer klinischer Bedeutung sind die Veränderungen der Epidermis, die eine komplexe Reduktion der Barrierefunktion und Minderung der Kompensationskapazität bezüglich exogener Noxen nach sich ziehen. Daraus leitet sich eine erhöhte Suszeptibilität insbesondere gegenüber Infektionen und Tumorerkrankungen ab. Vor diesem Hintergrund ist eine prophylaktische Strategie zur Substitution der physikochemischen und damit auch mikrobiologischen Barriere im Rahmen der Basispflege von großer Bedeutung. Um diese konsequent umsetzen zu können, ist die Empfehlung von explizit für Altershaut konzipierten Präparaten und praktischen Anwendungshinweisen sehr wesentlich. Letztere sollten die Einschränkungen bezüglich der Beweglichkeit sowie mögliche kognitive Defizite von Hochaltrigen berücksichtigen. Dazu sollten sowohl Eincremehilfen als auch bezüglich der Viskosität und Zusammensetzung geeignete Präparationen empfohlen werden. Um die Umsetzung zudem zu erleichtern, können schriftliche oder bildliche Handlungsempfehlungen sowie digitale Assistenzsysteme zur Anwendung kommen. Aufgrund der demografischen Entwicklungen in Deutschland und Europa wird die geriatrische Dermatologie in den nächsten Jahren deutlich an klinischer Relevanz gewinnen.

## Abstract (en)

Changes related to aging affect all layers of the skin and are influenced by both intrinsic conditions and extrinsic factors. The extent of the senescent changes can vary enormeously in seniors, so that an individual assessment is useful and often necessary. Of particular clinical importance are changes in the epidermis, which entail a complex reduction of the barrier function and a reduction in the compensatory capacity with regard to exogenous noxae. This results in increased susceptibility, especially toward infection and cancer. Against this background, a prophylactic strategy for the substitution of the physicochemical and thus also the microbiological barrier in the context of basic care is very important. In order to be able to implement these consistently, recommendations for preparations explicitly designed for aging skin as well as practical instructions for use are highly meaningful. The latter should take into account limitations regarding mobility as well as possible cognitive deficits of seniors. For this purpose, creams and suitable preparations in terms of viscosity and composition should be recommended. In order to facilitate implementation, written or pictorial recommendations for application as well as digital assistance systems can be used. Due to demographic developments in Germany and Europe, the clinical relevance of geriatric dermatology will significantly increase in the future.

Die demografische Entwicklung in Deutschland, die eine zunehmende Alterung der Bevölkerung ausweist, stellt unsere Gesellschaft vor eine große Herausforderung [1]. Dies wird besonders im Gesundheitswesen deutlich, da in allen Fachdisziplinen vermehrt hochaltrige Patient:innen versorgt werden müssen [2]. Man spricht auch von einer Geriatrisierung der Medizin. Für die Dermatologie bedeutet dies, nicht nur die besonders häufigen Erkrankungen des Hautorgans im Alter (z. B. Tumoren, Infektionen) im Fokus zu haben, sondern die besonderen Pflegebedürfnisse der seneszenten Haut zu erkennen und zu berücksichtigen [3]. Aufgrund der nach außen sichtbaren Anzeichen der Hautalterung und der sozialen Bedeutung, die der Haut zukommt, ist nicht nur im europäischen Kulturkreis der Wunsch nach einer Reduktion der Sichtbarkeit von Alterung stark verankert. Antiaging-Produkte werden deshalb häufig angewendet und besitzen eine große Bedeutung im Kosmetikmarkt [4–6]. Nicht zuletzt aufgrund der regulatorischen Rahmenbedingungen ist die Evidenz der von diesen Produkten vermittelten Effekte häufig sehr begrenzt [7]. Aussagen zu ausgelobten Effekten sind deshalb sehr stark Marketing-getriggert und sollten präparatebezogen hinterfragt werden.

Aus medizinischer Sicht viel relevanter als die soziale Bedeutung sind die seneszenzbedingten Einschränkungen der Regulationsbreite und Resilienz der epidermalen Barriere insbesondere bezüglich der physikochemischen Barrierefunktion [7]. Diese sind wesentlich von intrinsischen Faktoren abhängig, können aber durch extrinsische Einflüsse erheblich verstärkt werden. Dies führt zu einem sehr individuell geprägten klinischen Phänotyp des Barriereschadens und bildet die Grundlage für das gehäufte Auftreten insbesondere von Infektionen, Ekzem- und Tumorerkrankungen des Hautorgans im Alter. Um der Entstehung dieser Altersdermatosen prophylaktisch entgegenzuwirken, wird die Anwendung einer intensivierten barriereprotektiven Hautpflege empfohlen, die durch Substitution von Wasser, wasserbindenden Faktoren und insbesondere membranbildenden Lipiden die Suszeptibilität der Haut gegenüber exogenen Barrierenoxen reduzieren soll. Darüber hinaus sollte zur Primär- oder Sekundärprophylaxe insbesondere von aktinischen Plattenepithelkarzinomen die Anwendung von Sonnenschutzmitteln propagiert werden. Dabei ist aber eine regelmäßige, mindestens 2‑mal tägliche, Anwendung notwendig, um relevante Effekte zu erzielen. Hochaltrige Menschen können zwar häufig zur Anwendung einer Basispflege motiviert werden, sind aber durch orthopädische und häufig auch kognitive Einschränkungen auf pflegerische Hilfe bzw. Assistenz angewiesen [8]. Dies gilt darüber hinaus auch für die Anwendung von topischen Arzneimitteln zur Behandlung etablierter Altersdermatosen. Vor diesem Hintergrund ist eine Analyse der Zusammenhänge, die Definition von Problemsituationen und die Erarbeitung von Lösungsansätzen notwendig und im Rahmen einer optimierten dermatologischen Betreuung sowohl prophylaktisch als auch therapeutisch zwingend geboten.

## Demografische Entwicklung

Der demografische Wandel ist auch in Deutschland in vollem Gange, die Bevölkerung altert immer schneller. Dies bedeutet eine große Herausforderung für die sozialen Sicherungssysteme. Im Jahr 2020 waren bereits rund 22 % der Deutschen über 65 Jahre alt, womit Deutschland im europäischen Vergleich an fünfter Stelle steht [9]. Vorausberechnungen gehen davon aus, dass 2060 bereits 31 % der Bevölkerung über 65 Jahre alt sein werden [10]. Der Altenquotient, der angibt, wie viele über 65-Jährige auf 100 Personen im erwerbsfähigen Alter kommen, lag 2020 bei 37, im Jahr 2060 wird er voraussichtlich schon bei 60 liegen. Die ostdeutschen Bundesländer sind von der Überalterung der Gesellschaft noch stärker betroffen als die westdeutschen. In einigen der ostdeutschen Bundesländer liegt der Altenquotient bereits deutlich über dem Bundesdurchschnitt.

Die Lage spitzt sich aktuell insgesamt dadurch zu, dass die sog. „Baby-Boomer-Generation“ ins Rentenalter eintritt. Dies sind die geburtenstarken Jahrgänge der Nachkriegszeit, die nun in einer Art großen Welle aus der Erwerbstätigkeit ausscheiden. Das Risiko, pflegebedürftig zu werden, steigt mit dem Alter bekanntlich an. Im Jahr 2021 waren in Deutschland im Sinne des Pflegeversicherungsgesetzes (SGB [Sozialgesetzbuch] XI) 4,96 Mio. Menschen insgesamt und 3,63 Mio. Hochaltrige (> 70 Jahre) pflegebedürftig. Der Anteil pflegebedürftiger Personen nimmt ab dem 60. Lebensjahr exponentiell zu und erreicht mit dem 80. Lebensjahr eine Pflegequote von 25/35 % (Männer/Frauen) und ab dem 90. Lebensjahr von 70/87 % (Männer/Frauen). Etwa 80 % aller Pflegebedürftigen werden zu Hause durch Angehörige oder ambulante Pflegedienste versorgt. Die restlichen 20 % leben vollstationär in Pflegeeinrichtungen [11]. Zu den Aufgaben, die durch die Pflegenden übernommen werden, gehört oft auch die komplette Hautpflege.

Daten der Kassenärztlichen Bundesvereinigung zeigen, dass auch die dermatologischen Patienten überwiegend zur Gruppe der Hochaltrigen gehören. Im Jahr 2018 waren 52,4 % der stationär behandelten dermatologischen Patienten bereits über 65 Jahre alt und 35,3 % bereits über 75 [12]. Dabei handelt es sich in diesem Fachbereich selten um letale Krankheiten. Die dermatologischen Erkrankungen belegen jedoch laut der „Global Burden of Disease“-Studie weltweit den vierten Platz bezüglich des Leidensdrucks und der damit verbundenen Einschränkung der Lebensqualität [13, 14]. Um die steigenden Fallzahlen in der Dermatologie, die zum großen Teil durch Hochaltrige bedingt werden, zu bewältigen, ist es nötig, das Personal und alle, die in die Versorgung eingebunden sind, bezüglich der besonderen dermatologischen Bedürfnisse von Hochaltrigen zu schulen und zu sensibilisieren. Darüber hinaus werden in Zukunft zwangsläufig weitere Maßnahmen und Anstrengungen nötig sein, um den Erfordernissen einer qualitativ hochwertigen und den Menschen zugewandten Pflege gerecht zu werden.

## Grundlagen der Hautalterung

Wie jedes Organ des menschlichen Körpers ist auch die Haut unausweichlich vom Alterungsprozess betroffen. Dabei unterscheidet man zwischen der intrinsischen und extrinsischen Hautalterung. Die intrinsische oder endogene, chronologische Alterung ist zeitabhängig und wird beeinflusst durch genetische, hormonelle und metabolische Faktoren [15]. Besonders sichtbar werden die Zeichen der intrinsischen Hautalterung in lichtgeschützten Arealen wie den Oberarminnenseiten oder der Glutealregion. Klinisch imponiert die endogen gealterte Haut als feinfaltig, trocken und atroph [16]. Die extrinsische Alterung ist hingegen v. a. verhaltensabhängig. Sie kann als eine Superposition der endogenen Alterung verstanden werden [17], da die intrinsischen Mechanismen durch Umwelteinflüsse wie ultraviolette und Infrarotstrahlung, Rauchen und Luftverschmutzung verstärkt werden. Den größten Einfluss hat dabei das UVA-Licht, weshalb man auch von Lichtalterung spricht.

Der Seneszenz liegen verschiedene zelluläre Mechanismen zugrunde. Die Grundlage bildet eine Abnahme der zellulären DNA(Desoxyribonukleinsäure)-Reparaturkapazität und daraus resultierend eine Anhäufung von DNA-Mutationen, insbesondere der mitochondrialen DNA, sowie eine Telomerverkürzung bzw. Telomeraseinsuffizienz, die im Rahmen der Zellteilung zum Tragen kommt. Darüber hinaus induziert eine vermehrte Bildung von reaktiven Sauerstoffspezies zellulären oxidativen Stress und bedingt eine Aktivierung von Signalwegen, die zur vermehrten Bildung von kollagendegradierenden Matrixmetalloproteinasen führt. Dies führt in den verschiedenen Zellsystemen der Haut zur Abnahme des Kollagengehaltes, der Hautfeuchtigkeit und der Talgproduktion sowie zu einer veränderten Hautdicke [16]. Dabei werden ein atropher und ein hypertropher Phänotyp beschrieben, die sich histologisch wesentlich durch das Ausmaß der solaren Elastose unterscheiden [17].

Somit treten in allen 3 Hautschichten Veränderungen auf. Durch die Umverteilung des Fettes im Körper nimmt das subkutane Fettgewebe zugunsten des Stammfetts ab. Dies wird besonders an Händen, Füßen und im Gesicht sichtbar. Auch in der Dermis und Epidermis kommt es zu einem atrophischen Umbau. Die kollagenen und elastischen Fasern werden von Matrixmetalloproteinasen (MMPs) vermehrt abgebaut, und es kommt durch fragmentiertes Kollagen zu einer Hemmung der Neokollagenese, also einem positiven Feedbackmechanismus, der zur weiteren Degradation der Extrazellulärmatrix beiträgt [15]. Die physiologischen Gegenspieler der MMPs, die „tissue inhibitors of matrixproteinases“ (TIMPs), weisen hingegen eine verminderte Aktivität auf [18]. In der Dermis nehmen zudem sowohl Anzahl als auch Aktivität von Talg- und Schweißdrüsen ab, was zu einer verminderten Sebumproduktion und zu erschwerter Thermoregulation führt [19]. Letzteres wird zudem durch die Abnahme der Anzahl der Kapillaren und eine erhöhte Gefäßfragilität verstärkt [20]. Der Umbau im korialen Fasergerüst und das dadurch bedingte Abflachen der Reteleisten in der dermoepidermalen Junktionszone bedingen eine erhöhte mechanoelastische Anfälligkeit gegenüber Scherkräften. Auch auf epidermaler Ebene kommt es durch eine verringerte proliferative Aktivität von Keratinozyten zu einer Atrophie, die insbesondere das Stratum spinosum betrifft [21]. Zudem finden sich im Stratum basale als Zeichen einer nachhaltig gestörten Regeneration zunehmend dysplastische Keratinozyten. Letztlich münden diese epidermalen Veränderungen in eine Differenzierungsstörung des Stratum corneums; im Detail einer verminderten Zahl von Korneozyten und einem sowohl reduzierten Gehalt als auch Spektrum von Neutrallipiden und natürlichen Feuchthaltefaktoren was letztlich zu einer komplex gestörten physikochemischen Barrierefunktion führt [7, 22]. Klinisch sichtbar wird eine sich daraus ableitende Reduktion der Wasserbindungskapazität durch die Ausbildung des Symptoms „trockene Haut“. Zudem zeigen sich ein Anstieg des Hautoberflächen-pH-Werts, eine Verschiebung des kornealen pH-Gradienten und eine Abnahme der Pufferkapazität des Stratum corneum. Dies wiederum verstärkt die metabolische Degradierung der Barrierelipide, insbesondere der Ceramiden, bedingt eine Abnahme der antimikrobiellen Peptide sowie der Diversität der kutanen Mikrobiota und stört zudem nachhaltig die immunologische Funktionalität der Haut. Letztere wird durch weitere zelluläre Funktionsdefizite von Immunzellen im Rahmen des sog. „Inflammaging“ verstärkt, sodass eine komplexe Immundefizienz des Hautorgans resultiert [20].

## Einflussfaktoren auf Hautveränderungen im Alter

**Figure 1:**
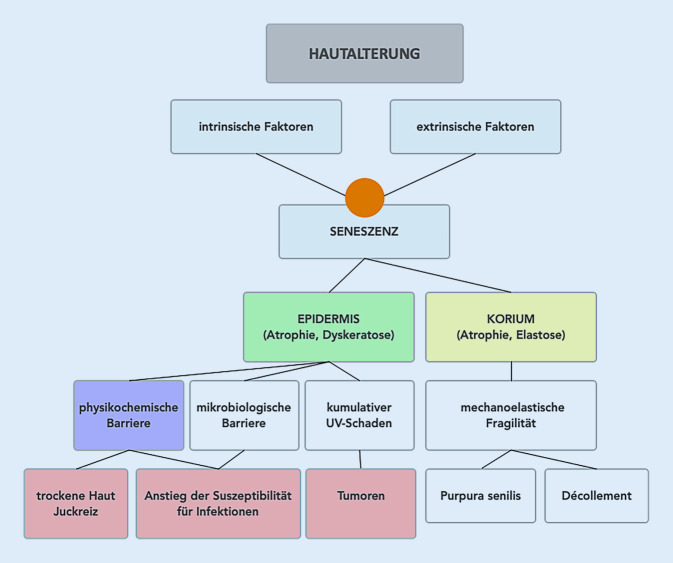

Aufgrund der funktionellen Defizite in physiologischen Abläufen der Altershaut wird diese suszeptibel für pathologische Veränderungen (Abb. 1; [23]). Dies kann durch die Einnahme bestimmter Arzneimittel (z. B. Statine, Gliptine, Immunsuppressiva) bzw. durch das Vorliegen von altersbedingter Komorbidität potenziert werden [24]. Die konkreten, klinisch relevanten Einschränkungen können vielfältig sein und sind individuell unterschiedlich [14]. Dabei treten v. a. vermehrt benigne und maligne Tumoren sowie Infektionskrankheiten auf [25]. Besonders häufig zeigen sich dabei Mykosen, aber auch bakterielle oder virale Infektionen. Von besonderer klinischer Relevanz, insbesondere bei bettlägerigen oder immobilen Patient:innen, sind lagerungsbedingte Druckläsionen [3]. Zudem treten häufig inkontinenzassoziierte irritative Kontaktekzeme auf, die durch die erhöhte Suszeptibilität der Altershaut besonders ausgeprägt sein können. Ein Großteil der Hochaltrigen klagt über Xerosis cutis und den damit einhergehenden Pruritus. Dieser kann zu Kratzexkoriationen führen, die wiederum als Eintrittspforte für Infektionserreger dienen und aufgrund der verzögerten Wundheilungsbedingungen persistieren können. Die in der Literatur angegebene Prävalenz der Xerosis cutis bzw. des altersassoziierten Pruritus variiert stark. Als relevante Augmentationsfaktoren gelten renale bzw. hepatische Funktionseinschränkungen [26]. Zudem kann die Verfügbarkeit einer biokompatiblen Wasserphase im Stratum corneum durch eine Umverteilung bei Anwendung z. B. von Diuretika reduziert werden [8].

In vielen Ländern ist die Xerosis cutis das häufigste dermatologische Problem in der geriatrischen Population. Die Zahlen variieren zwischen 5,4 % bis zu 85,5 % [27, 28]. In Deutschland wurde unter Pflegeheimbewohnern sogar eine Prävalenz von 99 % für Xerosis cutis ermittelt [29]. Pruritus wurde in diesem Zusammenhang mit einer Prävalenz von 8,8–48,5 % beobachtet [27, 30].

Mykotische Hautinfektionen treten mit einer Prävalenz von 10,4–64 % auf [26, 26, 31]. Die häufigsten Manifestationsformen sind dabei Dermatophytosen (Onychomykosen und Tinea pedis) sowie Hefepilzinfektionen, allem voran intertriginöse Kandidosen durch *Candida albicans*. Die Prävalenz von Infektionen durch Bakterien und Viren reicht von 2,8–12,3 % [27]. Von Dekubiti besonders betroffen sind bettlägerige Patienten. Sie treten in Deutschland bei 1 % der Krankenhauspatienten und bei bis zu 9 % der Pflegeheimbewohner auf [29]. In den USA reichen die Zahlen bis zu 30 % und in Kanada sogar bis zu 46 % der Pflegeheimbewohner. Die inkontinenzassoziierte Dermatitis wird in den USA mit einer Prävalenz von 3,5–22,6 % beobachtet [32, 33]. In Deutschland liegt die Prävalenz hingegen bei 35,4 % [29].

## Altersgerechte Basispflege

Die strukturellen und funktionellen Veränderungen in der seneszenten Haut und die damit verbundenen Funktionsveränderungen erfordern eine prophylaktische, ggf. auch therapeutische barriereprotektive Hautpflege [34]. Das Konzept der Anwendung dieser kosmetischen Mittel, stofflichen Medizinprodukte oder arzneistofffreien Arzneimittel basiert auf den Erkenntnissen, welche Komponenten der physikochemischen Barriere qualitativ oder quantitativ vordergründig in der seneszenten Haut defizitär sind. Dabei sind für den Aufbau von Membranstrukturen in der interkorneozytären Matrix, die das morphologische Äquivalent für das darstellen, was als physikochemische Barriere bezeichnet wird, membranbildende Lipide (Ceramide bzw. Phospholipide), Wasser und hygroskopische Moleküle die zentral bedeutenden Substituenten. Besonders relevant erscheint die Zusammensetzung der hydrophilen Substitutionsphase zu sein. So sind die Wahl eines sauren pH-Wertes (≤ 5,5), einer hohen Pufferkapazität sowie die Wahl inerter und effektiver Humectants, wie z. B. Glycerol oder Urea, wichtige Faktoren für eine effektive Rehydrierung [35]. Außerdem sind die mindestens 2‑mal tägliche Applikation der Basispflege sowie die Umsetzung allgemeiner Verhaltensregeln von Bedeutung. Dazu zählen das Vermeiden von zu langem und zu heißem Duschen oder Baden, die Verwendung von synthetischen Detergenzien statt Seifen und das Vermeiden von zu trockener Raumluft [36]. Zudem ist auf ausreichende Flüssigkeitszufuhr zu achten, da im Alter sowohl das Durstgefühl reduziert ist, als auch Primärtherapien mit Diuretika zu einem zusätzlichen Flüssigkeitsverlust führen können bzw. Statine durch Reduktion der keratinozytären Lipidsynthese die physikochemische Barriere des Stratum corneum reduzieren.

Eine Besonderheit der topischen Therapie im Alter sind die veränderten Diffusionsbedingungen der seneszenten Haut. Bei der Anwendung von topischen Arzneimitteln sollte bedacht werden, dass die Wirkstoffaufnahme durch die Atrophie des Stratum corneum (Reservoirfunktion) vermindert, aber gleichzeitig die epitheliale Permeationsrate gegenüber adulter Haut erhöht sein kann [37]. Zudem ist von einer Änderung der Diffusionsbedingungen durch die verminderte Mikrozirkulation im korialen Gewebe auszugehen [37]. Aus praktischer Sicht sollte zudem bedacht werden, dass Personen, die älter als 65 Jahre sind, nahezu regelmäßig aus klinischen Studien zur Testung von Arzneimitteln, Medizinprodukten oder Kosmetika ausgeschlossen werden [38]. Somit gibt es für die Empfehlungen zum Einsatz und zur Sicherheit von Topika bei Senioren eine eher geringe Evidenz [39]. Vorliegende Daten zu den gängigen Parametern für die Bewertung der physikochemischen Barrierefunktion bei Hochaltrigen sind zudem uneinheitlich. Dies lässt zunächst auf eine Inhomogenität der pathophysiologischen Faktoren an sich, aber auch auf Unterschiede an den für die Messung benutzten Körperarealen bzw. auf Unterschiede im Ausmaß der Barrierestörung in Altersgruppen innerhalb der Hochaltrigenpopulation schließen. Darüber hinaus sollte bedacht werden, dass sowohl die Messung des totalen Wassergehaltes des Stratum corneum (Corneometrie) als auch die Messung des transepidermalen Wasserflusses (Tewametrie oder Evaporimetrie) lediglich eine indirekte Bewertung der physikochemischen Barriere, also der Membranfunktionalität, zulassen. Dies begründet auch, dass einige Studien eine Erniedrigung der Tewametriewerte bei Hochaltrigen zeigen [40]. Von den Autoren wird dies zudem uneinheitlich bewertet und entweder als Barrieredefizit oder als Barrieresteigerung interpretiert [35, 37, 40–43]. Deshalb ist davon auszugehen, dass die Tewametrie alleinig nicht als Parameter zur Bewertung geeignet ist. Erst die Kombination von Untersuchungen des Lipidspektrums des Stratum corneum, der Quantität der Bestandteile des natürlichen Feuchthaltefaktors und funktioneller Barriereparameter scheinen den komplexen Veränderungen gealterter Haut gerecht zu werden [44]. Dennoch besteht grundsätzlich am Funktionsdefizit der physikochemischen Barriere im Senium kein Zweifel [45–47].

Weniger untersucht und konsistent sind Aussagen zur kutanen Mikrobiota der seneszenten Haut [48]. Dies liegt v. a. an den bisher grundsätzlich bestehenden methodischen Mängeln bei Studien und Nachweisverfahren zum kutanen Mikrobiom sowie dessen stark interindividuell variierender Diversität. Die Zunahme von Infektionserkrankungen der Haut im Alter lässt zwar relevante Änderungen als sehr wahrscheinlich erscheinen, diese können aber aktuell nicht seriös näher belegt werden. Bestimmte Umstände wie Okklusion (z. B. durch Hautfalten oder Inkontinenztextilien) oder Hyperhydratation (verändertes Schwitzverhalten oder Mazeration) könnten zusätzliche Einflussfaktoren auf die mikrobiologische Barriere sein [8].

Der professionelle Einsatz von spezifisch für die Altershaut konfigurierten Formulierungen kann effektiv der erhöhten Prävalenz von Altersdermatosen entgegenwirken [29, 35, 49].

## Adhärenz bei Hochaltrigen

Grundlage einer Förderung der Adhärenz von Hochaltrigen zu Maßnahmen der altersgerechten prophylaktischen Basispflege bzw. zur topischen Therapie von Hauterkrankungen ist ein klar kommuniziertes, strukturiertes und unkompliziertes Empfehlungsregime. Die ärztlichen Empfehlungen sollten deshalb zunächst das Verständnis für die Notwendigkeit prophylaktischer oder therapeutischer Maßnahmen in einfachen Worten und logischen Zusammenhängen ins Zentrum der Kommunikation stellen. Gleichzeitig sollte aktiv die Durchführbarkeit der Empfehlungen erörtert und neben der selbstständigen Umsetzung sollten auch Möglichkeiten der Assistenz aktiv angesprochen und hinterfragt werden. Dabei sollte der empfehlende Arzt eine Einschätzung der Beweglichkeit bzw. orthopädischer Einschränkungen sowie die kognitiven Voraussetzungen für eine Umsetzung bedenken [50]. Der Wille zur Adhärenz ist bei Hochaltrigen in der Regel sehr hoch, allerdings sind die realistische Selbstreflexion und die Fähigkeit zum Antizipieren von Prozeduren eingeschränkt [51]. Um diese Zusammenhänge zu objektivieren, können verschiedene Assessments und Tests, die die Autonomie und Aktivität von Senioren messen, eingesetzt werden. Hierzu zählen die Bestimmung der „activities of daily living“ (ADL) nach Katz sowie der Barthel-Index [52]. Mit der ADL können die individuellen Fähigkeiten zur selbstständigen Umsetzung alltäglicher Aktivitäten bei hochaltrigen Personen charakterisiert werden. Man unterscheidet die Basis-ADL (BADL) und die instrumentellen ADL (IADL) [53]. Die BADL umfassen Tätigkeiten wie Toilettennutzung, Duschen und Baden sowie An- und Auskleiden. Die IADL hingegen beziehen sich auf komplexere Tätigkeiten wie die Verwaltung der Eigenmedikation oder Finanzen. In Bezug auf dermatologisch relevante Aktivitäten kann durch Ähnlichkeiten in Bewegungsabläufen beim Waschen, Abtrocknen und Eincremen anhand der BADL geschätzt werden, welche objektivierbaren Einschränkungen der Hautpflege vorliegen.

In der Europäischen Union sind laut Daten des Robert Koch-Instituts in der Altersgruppe der über 65-Jährigen im Durchschnitt 8,4 % bei den BADL und 25,2 % bei den IADL eingeschränkt [54]. Der Durchschnitt der ADL-Einschränkung bei Hochaltrigen in Deutschland liegt etwas niedriger (6,3 % BADL und 14 % IADL). Frauen, Personen über 75 Jahre und Senioren mit niedrigem Bildungsniveau sind im Durchschnitt stärker von den Einschränkungen bei den IADL betroffen. Bei Hochaltrigen mit einer leichten kognitiven Beeinträchtigung ist zudem die Wahrscheinlichkeit, bei den alltäglichen Aktivitäten eingeschränkt zu sein, erwartungsgemäß erhöht [50]. Dabei ist von einer leichten kognitiven Beeinträchtigung auszugehen, wenn diesbezüglich Defizite vorliegen, die über die Altersnorm hinausgehen, aber die Kriterien einer Demenz nicht erfüllen [55]. Bei der Betrachtung von kognitiven Defiziten ist es wichtig, das Ausgangsniveau der Intelligenz zu berücksichtigen und ein Bias durch depressive Verstimmungen auszuschließen. Außerdem haben Hochaltrige mit Einschränkung der ADL auch ein erhöhtes Risiko für motorische Einschränkungen durch verminderte Muskelkraft, den Abbau von Muskelmasse und das Nachlassen der körperlichen Performance [56]. Dies verstärkt wiederum im Umkehrschluss die Einschränkungen bei den ADL.

Da sich der körperliche und kognitive Abbau im Alter gegenseitig verstärken, ist ein ganzheitliches Konzept zur Therapie und Prävention nötig. In Studien zur Adhärenz von Hochaltrigen hat sich gezeigt, dass eine Reduktion der Anzahl an Medikamenten bzw. therapeutischen Interventionen (wenn medizinisch vertretbar) sehr sinnvoll ist, ebenso wie das Aushändigen von schriftlichen Anweisungen, Erinnerungshilfen bzw. Strategien zur Patientenedukation [51, 57].

## Ausblick und Handlungsempfehlungen

Um die Autonomie im Alter möglichst lange zu erhalten, gilt es, Strategien zu entwickeln, welche die selbstständige Hautpflege für Hochaltrige erleichtern. Dazu sind klare Konzepte notwendig, die einfach, verständlich und durchführbar sind.

Da es bisher oft gängige Praxis ist, Hochaltrige über 65 Jahre aus klinischen Studien auszuschließen, liegt ein Mangel an repräsentativen Daten für diese Personengruppe vor [38]. So besteht ein großer Bedarf, Daten zu generieren, die den tatsächlichen Zustand der Haut nicht nur von hospitalisierten Hochaltrigen oder Pflegeheimbewohnern, sondern auch von in der Häuslichkeit lebenden und sich selbst versorgenden Personen der Altersgruppe zu erfassen. Darüber hinaus wäre es angezeigt, entsprechende Daten der Hautpflege bei Hochaltrigen grundsätzlich auch in der Pflegedokumentation im ambulanten und stationären Bereich zu erfassen. Dabei wäre es sehr vorteilhaft, wenn einfache technische Messgeräte verfügbar wären, um standardisiert definierte Parameter der physikochemischen Barrierefunktion routinemäßig zu erfassen und den individuellen Nutzen einer Intervention objektivieren zu können.

Zudem sollten Maßnahmen und Strategien der Unterstützung bei der eigenständigen Hautpflege sowohl an die Hochaltrigen selber als auch an die Pflegenden kommuniziert, weiterentwickelt und bezüglich der Effektivität validiert werden. Aus heutiger Perspektive sind hier v. a. mechanische Eincremehilfen, insbesondere Stabroller, zur Applikation an schwer erreichbaren Körperarealen und technische Geräte zur Erinnerung an die Intervention zu nennen. Für die Zukunft sollten v. a. Möglichkeiten der digitalen Assistenz entwickelt und erprobt werden. Hierbei bieten digitale Gesundheitsanwendungen (DiGAs) eine besondere Möglichkeit, da diese datensicherheitstechnisch überprüft, mit Bezug auf einen positiven Versorgungseffekt regulatorisch validiert und erstattungsfähig sind. Zudem sollten bereits bei der Entwicklung von Hautpflegeprodukten für Hochaltrige bestimmte Produkterfordernisse berücksichtigt werden. So kann durch die Wahl geeigneter Primärpackmittel die Entnahme der Formulierung auch bei Kraftminderung oder Arthralgie der Hände ermöglicht werden. Die Verteilung der Formulierung auf der Hautoberfläche kann zudem durch niedrigvisköse Produkteigenschaften bzw. durch Rüsselaufsätze oder Schaumspender verbessert werden.

Auch kann durch eine gezielte galenische Formulierung die Hautpflege durch Minimierung der Rückstandphase die Anwendung erleichtern und die Unfallgefahr nach der Intervention reduzieren.

## Fazit für die Praxis

- Aufgrund der demografischen Entwicklungen in Deutschland und Europa wird die geriatrische Dermatologie in den nächsten Jahren deutlich an klinischer Relevanz gewinnen.
- Das Ausmaß der seneszenten Veränderungen kann bei Hochaltrigen stark variieren, sodass eine individuelle Bewertung sinnvoll und häufig auch notwendig ist.
- Von besonderer klinischer Bedeutung sind die Veränderungen der Epidermis, die eine komplexe Reduktion der Barrierefunktion und Minderung der Kompensationskapazität bezüglich exogener Noxen nach sich ziehen.
- Eine prophylaktische Strategie zur Substitution der physikochemischen und damit auch mikrobiologischen Barriere im Rahmen der Basispflege ist von großer Bedeutung.
- Um die Autonomie im Alter möglichst lange zu erhalten, gilt es, Strategien zu entwickeln, welche die selbstständige Hautpflege für Hochaltrige erleichtern.
